# Influence of Normal-to-High Anodizing Voltage on AAO Surface Hardness from 1050 Aluminum Alloy in Oxalic Acid

**DOI:** 10.3390/mi15060683

**Published:** 2024-05-23

**Authors:** Chin-An Ku, Chen-Chieh Wu, Chia-Wei Hung, Chen-Kuei Chung

**Affiliations:** Department of Mechanical Engineering, National Cheng Kung University, Tainan 701, Taiwan

**Keywords:** anodic aluminum oxide, AAO, hybrid pulse anodization, HPA, high hardness, hard anodization, rapid growth

## Abstract

Anodic aluminum oxide (AAO) has been widely applied for the surface protection of electronic component packaging through a pore-sealing process, with the enhanced hardness value reaching around 400 Vickers hardness (HV). However, the traditional AAO fabrication at 0~10 °C for surface protection takes at least 3–6 h for the reaction or other complicated methods used for the pore-sealing process, including boiling-water sealing, oil sealing, or salt-compound sealing. With the increasing development of nanostructured AAO, there is a growing interest in improving hardness without pore sealing, in order to leverage the characteristics of porous AAO and surface protection properties simultaneously. Here, we investigate the effect of voltage on hardness under the same AAO thickness conditions in oxalic acid at room temperature from a normal level of 40 V to a high level of 100 V and found a positive correlation between surface hardness and voltage. The surface hardness values of AAO formed at 100 V reach about 423 HV without pore sealing in 30 min. By employing a hybrid pulse anodization (HPA) method, we are able to prevent the high-voltage burning effect and complete the anodization process at room temperature. The mechanism behind this can be explained by the porosity and photoluminescence (PL) intensity of AAO. For the same thickness of AAO from 40~100 V, increasing the anodizing voltage decreases both the porosity and PL intensity, indicating a reduction in pores, as well as anion and oxygen vacancy defects, due to rapid AAO growth. This reduction in defects in the AAO film leads to an increase in hardness, allowing us to significantly enhance AAO hardness without a pore-sealing process. This offers an effective hardness enhancement in AAO under economically feasible conditions for the application of hard coatings and protective films.

## 1. Introduction

Aluminum alloy is one of the most commonly used metals in the industry. To adapt to different working environments and provide protection for electronic components, surface treatment of aluminum metal is a crucial problem for development. The anodic aluminum oxide (AAO) technology is the most well-known method for surface protection and modification [1,2,3,4,5,6]. In contrast to the thin oxide film formed naturally on aluminum metal under ambient conditions with thickness less than ten nanometers, AAO can be produced at thicknesses ranging from nanometers to tens of micrometers, depending on controlled parameters in the anodization process [7,8,9,10]. AAO technology is currently utilized for surface protection in various products such as cellphones, computers, aircraft, and aerospace equipment. 

As a ceramic material, AAO is well-known for its outstanding resistance to scratching and corrosion, and numerous studies focus on surface hardness improvement [11,12,13,14,15,16,17]. For instance, Ateş et al. [13] achieved a hardness of 52–264 HV by controlling different AAO preparation parameters without a pore-sealing process. However, this value falls below the recognized hardness (>400 HV) required for 3C products. Additionally, the process time is long, ranging from 8 to 16 h, which does not meet the efficiency requirements for industrial preparation. Modification methods to achieve desired properties often involve not only controlling anodizing parameters for structural adjustments but also employing sealing techniques such as boiling-water sealing, oil sealing, or salt-compound sealing. These sealing methods lead to a reduction in porosity, effectively enhancing surface hardness. Abdel-Gawad et al. [14] explored sulfuric acid anodizing combined with nickel acetate sealing for AA2024, AA6061, and AA7075, achieving high hardness (>400 HV). The anodizing process time in this case was only 30 min, aligning with the industry’s need for efficient preparation. However, a drawback of nickel acetate sealing is the deposition of nickel and nickel acetate coloration on the surface, which is a significant disadvantage for many products that rely on the AAO pore characteristics for coloring. Consequently, this is one of the reasons why nickel acetate sealing is not currently used in the industry. AAO has established certain standards for surface protection and impact testing in the casings of 3C products and the aerospace industry. Traditionally, the preparation of AAO requires pore-sealing processes [11,14], annealing [15], or the use of highly toxic sulfuric acid [12,16] to achieve the desired specifications for hardness. Otherwise, it may fall slightly short of requirements [13,17]. These methods are associated with disadvantages such as time-consuming, complex processes and toxic solutions. In the current industrial landscape, there is a demand for low-cost, rapid processing and compatibility with coloring processes. Methods such as oil sealing [18], Ni-P [19] sealing, or Ni-B [20] sealing, involving extended processing time, are still not a suitable solution. Therefore, proposing a rapid and simple process that does not affect the color of AAO surface to enhance hardness is still worthy of further exploration.

On the other hand, a rapid and highly efficient method called hard anodization (HA) for producing AAO was proposed by Lee et al. in 2006 [21]. The mild anodization (MA) and HA were compared from AAO produced in oxalic acid solutions using traditional 40 V and high voltage of 100–150 V. This work reveals that AAO growth rates increased several tens of times under high voltage HA conditions. This phenomenon is now widely used to improve the efficiency of AAO production, with many scholars conducting in-depth studies [22,23,24,25]. However, the distinction between MA and HA is not merely in the difference in growth rates: the former exhibits a significant decrease in water content of 0.3–0.4 wt.% in MA, compared to 0.1 wt.% in HA, and carbon contents of 2.4 wt.% in MA, compared to 1.8 wt.% in HA [21], along with a more compact structure with fewer impurities. In AAO prepared using oxalic acid, the carbon element originates from oxalate anions, which is also the reason for the photoluminescence (PL) effect in AAO. The PL characteristics of AAO were initially reported by Yamamoto et al. in 1981 [26], with a stronger signal in the PL spectrum near 470 nm, which is attributed to the contribution of oxalate ions. Meanwhile, Huang et al. [27] suggested that the PL in AAO membrane originates from oxygen ion defect states (F center) within the AAO membrane, with emission peaks located at 413 nm and 430 nm. The PL effect of AAO has been explored in many studies [28,29,30,31], among which Zheng et al. [31] investigated the variation in PL intensity of AAO under different anodizing voltages. It was found that the PL intensity of AAO significantly decreases at high voltages of 130 V compared with traditional MA at 40 V [31]. This implies that, under HA conditions, there may be lower water content and fewer anions entering the AAO pore walls, resulting in different compositions within the AAO.

Here, we improved the traditional HA process using HPA to enhance the surface hardness of AAO. Based on the characteristics of HA mentioned above, we proposed using hybrid pulse anodization (HPA) method [8,32] to conduct anodization at different voltages to enhance the AAO surface hardness. The HA method helps reduce the ingress of anions and water content diffusion into the AAO pore walls, which could result in a denser arrangement of AAO structure, leading to a significant improvement in hardness. The PL characteristics of oxalate anions and oxygen vacancies are also discussed for the mechanism of AAO hardness improvement. Additionally, the HPA process can suppress Joule heating, allowing the AAO reaction temperature to rise to room temperature, thus addressing the drawback of the traditional HA process, which is limited to low temperatures. By utilizing the instantaneous growth of AAO with high current density, surface hardness is significantly enhanced, achieving high hardness (>400 HV) for protection on AA1050 without pore sealing. This method allows for quick preparation, providing advantages of low cost, a simple process, and high efficiency. Moreover, the porous characteristic is preserved, making it suitable for further applications.

## 2. Materials and Methods

**Figure 1:**
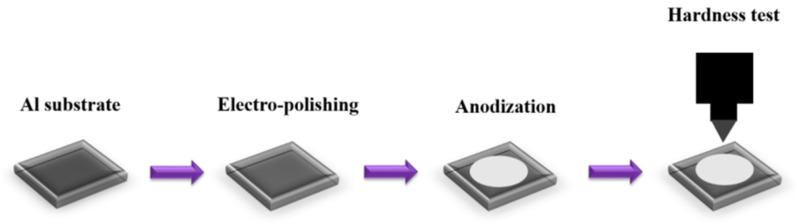
The experimental process flow for AAO hardness test.

The experimental process flow is drawn in Figure 1. First, the commercial 1050 aluminum alloy of 1 mm thick was cut into 2.5 × 2.5 cm^2^ for our experiments. The AA1050 was cleaned using DI water and electropolished by a solution of perchloric acid:ethanol = 1:1 (*v*/*v*) at 0 °C, at 20 V for 1 min, followed by perchloric acid:ethanol = 1:4 (*v*/*v*) for 5 min with the same parameters to reduce the surface roughness of commercial alloy. In terms of the anodization process, a hybrid pulse anodization (HPA) method [33,34,35] was employed for one-step anodization at 25 ± 0.5 °C in 0.3 M oxalic acid. The normal-to-high anodization potentials were set from normal (40 V) to high (100 V), with each sample controlled for various amounts of time to reach a thickness of 12 ± 0.5 µm, exploring changes in hardness under the same AAO thickness. The thickness of 12 µm was selected due to the current AAO thickness for 3C products is approximately 12 ± 1 µm. From the calculated growth rates and experiments, it was determined that it takes approximately 7200 s, 2520 s, 450 s, and 390 s, respectively, to achieve the specified thickness at voltages of 40 V, 60 V, 80 V, and 100 V. The duty ratio during the anodization process is controlled within 20~50% to avoid the burning effect from excessive Joule heating and preserve the pore structure. Microhardness testing was conducted using a Vickers hardness (HV) test, and the measurement results were averaged from 5 random points on each sample. The nanostructure of AAO was observed by high-resolution field scanning electron microscopy (HRFESEM, JEOL JSM-7000F, Tokyo, Japan), and the photographs were analyzed by using the commercial software ImageJ (ver. 1.53t) to investigate AAO pore size, porosity, and thickness. The PL spectrum is measured by a Micro-Raman and Micro-PL spectrometer (Jobin Yvon/Labram HR), with a laser wavelength of 325 nm.

## 3. Results and Discussion

**Figure 2:**
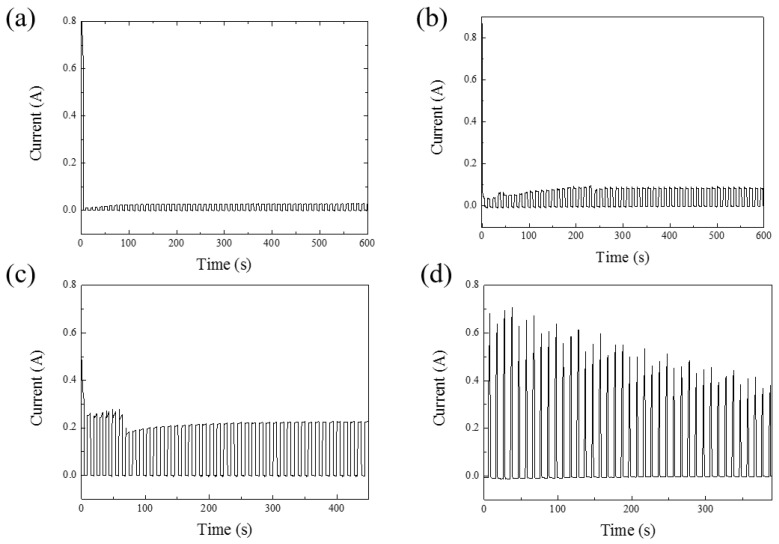
The current–time diagrams of AAO. (**a**) The first 10 min of AAO fabricated at 40 V. (**b**) The first 10 min of AAO fabricated at 60 V. (**c**) The whole process of AAO fabricated at 80 V for 450 s. (**d**) The whole process of AAO fabricated at 100 V for 390 s.

Figure 2a–d illustrates the current–time plots for anodization voltages ranging from 40 V to 100 V. Figure 2a,b depicts the initial 10 min of the reaction at 40 V and 60 V in HPA, showing that the current trend stabilizes at around 200 s. Figure 2c,d represents the entire process’s current–time relationship at 80 V and 100 V, respectively. Due to the capacitive characteristics of AAO, there is a slight reverse discharge effect when no voltage is applied. Therefore, in this experiment, a small negative voltage is applied to suppress this effect, aiming to reduce Joule heating in HPA and further preserve pore integrity [35]. In Figure 2a–d, almost zero current can be observed in the negative voltage period, illustrating the method of maintaining nanoscale pore integrity under high-voltage conditions at room temperature using HPA.

**Figure 3:**
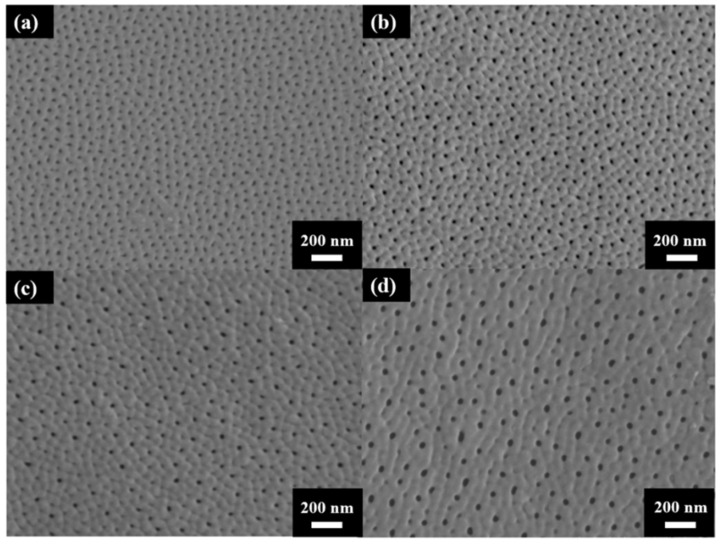
The top-view SEM images of AAO fabricated at (**a**) 40 V, (**b**) 60 V (**c**) 80 V, and (**d**) 100 V by the one-step HPA method at 25 °C.

**Figure 4:**
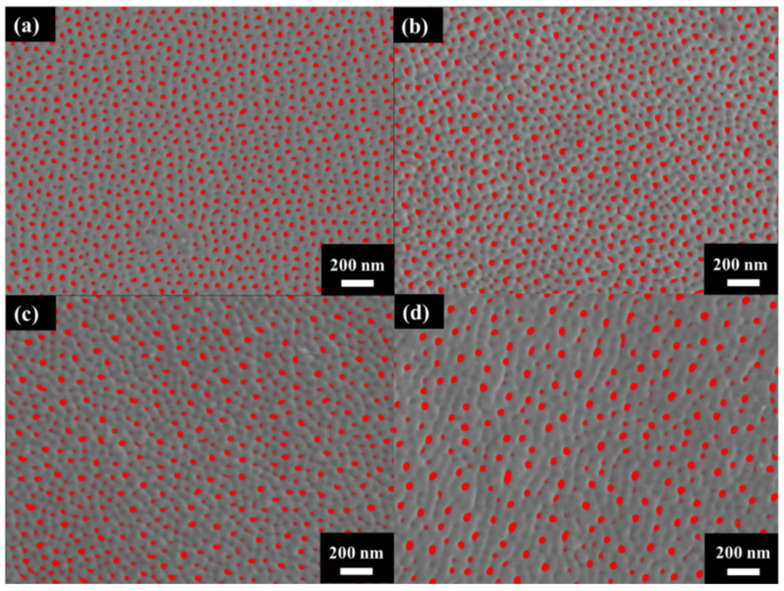
The gray-scale SEM micrographs for porosity analysis under anodization voltage at (**a**) 40 V, (**b**) 60 V (**c**) 80 V, and (**d**) 100 V.

Figure 3 shows the surface morphology of AAO prepared at (a) 40 V, (b) 60 V, (c) 80 V, and (d) 100 V. The AAO pore structure is observed to be complete and without signs of burning due to the unique HPA technique applied in our laboratory. By using a small negative potential after a large anodization positive potential, a regular period of 0 current during the anodization process is achieved. This allows AAO to dissipate heat and maintain a complete structure during growth. Through image analysis, the average pore diameters in the SEM images were calculated as 31.1 ± 4.0 nm, 33.1 ± 3.7 nm, 41.2 ± 3.7 nm, and 43.3 ± 3.8 nm, corresponding to anodization voltages at 40–100 V. In Figure 4, the porosities were investigated by the gray-scale image analysis, with results of 10.2 ± 0.9%, 9.1 ± 0.8%, 7.3 ± 0.6%, and 6.7 ± 0.5%. Although the pore diameter increases with voltage, the porosity of high-voltage AAO decreases due to the growth of interpore distance. Hence, there are fewer pores and a lower porosity in AAO prepared under high voltage conditions. This also indicates a higher proportion of AAO, which is conducive to the enhancement of hardness. 

**Figure 5:**
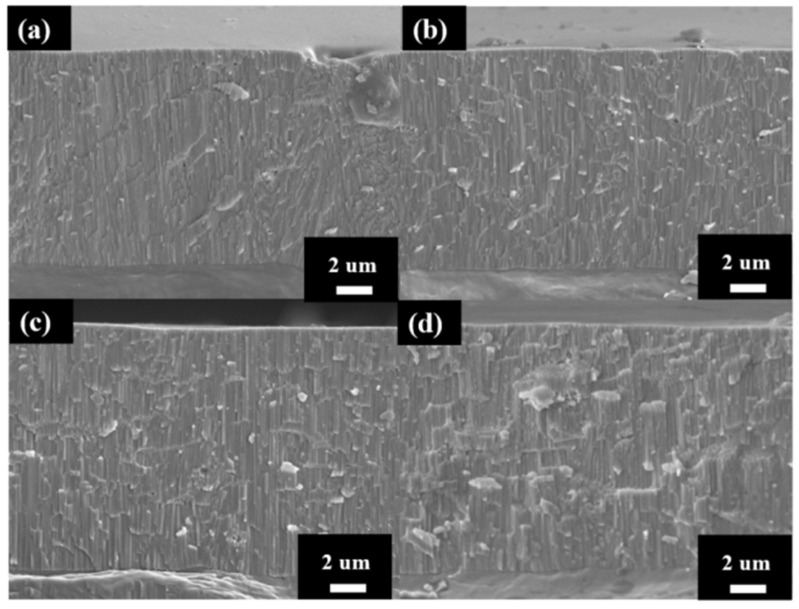
The AAO thickness at (**a**) 40 V, (**b**) 60 V (**c**) 80 V and (**d**) 100 V by one-step HPA method. The thickness was controlled at 12 ± 0.5 µm.

The cross-section micrographs of AAO thickness from controlling the growth at the same thickness under different potentials are shown in Figure 5. After multiple experiments, we finally conducted anodization for 2 h, 42 min, 8 min, and 6 min 30 s at 40 V, 60 V, 80 V, and 100 V, respectively, and achieved thicknesses of 11.9 ± 0.5, 12.2 ± 0.4, 12.1 ± 0.6, and 12.3 ± 0.7 µm. The anodization parameters and pore structure of AAO are also listed in Table 1 for comparison. Since thickness is the primary factor affecting AAO hardness, we controlled all thicknesses to be within 12 ± 0.3 µm to avoid the influence of this parameter. In addition, the growth rate of the 100 V sample reached 1.9 μm/min, faster than traditional mild or hard anodization methods, and also 18 times the rate for our 40 V sample without burning effect. This is attributed to the regular cooling mechanism in HPA, allowing the growth rate to surpass traditional limits and achieve faster AAO growth. However, there is little difference in the growth rates of AAO prepared at 80 V and 100 V, indicating that there is a substantial increase in growth rate once the voltage exceeds a certain degree. Additionally, as the voltage increases from 40 V to 100 V, the porosity of AAO decreases from 10.2% to 6.7%. This contributes to the increase in interpore distance with higher voltage. Porosity also represents the air content within the AAO film, so it is expected that high-voltage AAO with lower porosity will exhibit better hardness performance.

**Figure 6:**
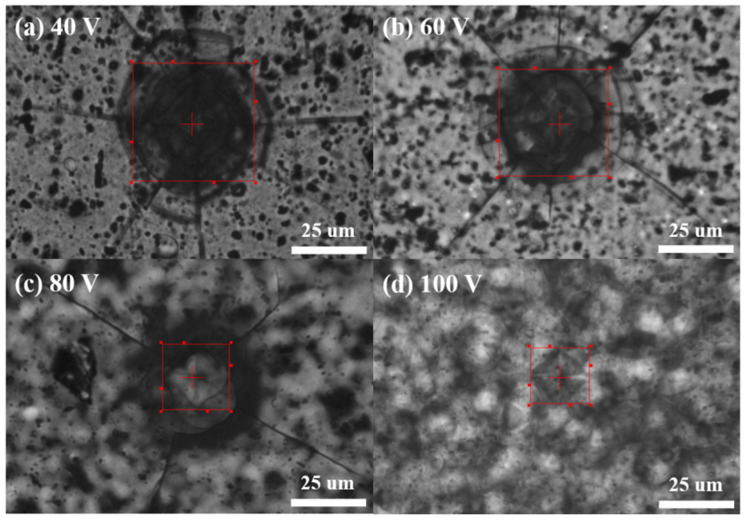
The AAO hardness from AAO fabricated at (**a**) 40 V, (**b**) 60 V, (**c**) 80 V, and (**d**) 100 V. The micro-hardness measurement results are 83 HV, 127 HV, 320 HV, and 423 HV, respectively.

Figure 6 presents the results of hardness testing on AAO samples, where the results for 40–100 V are (a) 83 HV, (b) 127 HV, (c) 320 HV, and (d) 423 HV. The measurement results were averaged from five random points on each sample, and we obtained similar results by repeating the experiment three times. We observed a significant improvement in the hardness of AAO with an increase in potential, even at the same thickness of 12 ± 0.3 µm. The hardness at 100 V is approximately five times higher than the traditional parameter at 40 V, with the most noticeable change observed in the experimental data from 60 V to 80 V. Therefore, elevating the potential in the anodization process not only effectively increases the growth rate of AAO but also enhances surface hardness. This provides an opportunity for AAO to achieve high hardness while retaining its porous characteristics, contributing to the potential for diverse applications. 

The increase in AAO hardness with the rise in voltage can be attributed to two reasons: first, the decrease in porosity from 10.2% to 6.7% with increasing voltage, as listed in Table 1; secondly, the reduction in water and carbon contents with voltage, corresponding to the photoluminescence (PL) spectrum. The structure factor to influence surface hardness is the porosity of AAO. While the pore diameter of AAO increases with voltage, the interpore distance also increases, leading to a slight decrease in porosity. Relevant data can be observed from SEM images and summarized in Table 1, where the porosity of AAO at 100 V is 6.7%, a 3.5% decrease compared to AAO at 40 V. This reduction in porosity decreases the air content, contributing to a minor increase in the surface hardness of AAO. However, the traditional DCA preparation method cannot achieve such rapid growth without a burning effect under high-voltage and room-temperature conditions. Therefore, employing HPA technology to increase the instantaneous growth rate of AAO is an effective way to enhance the hardness of AAO without a pore-sealing process.

**Figure 7:**
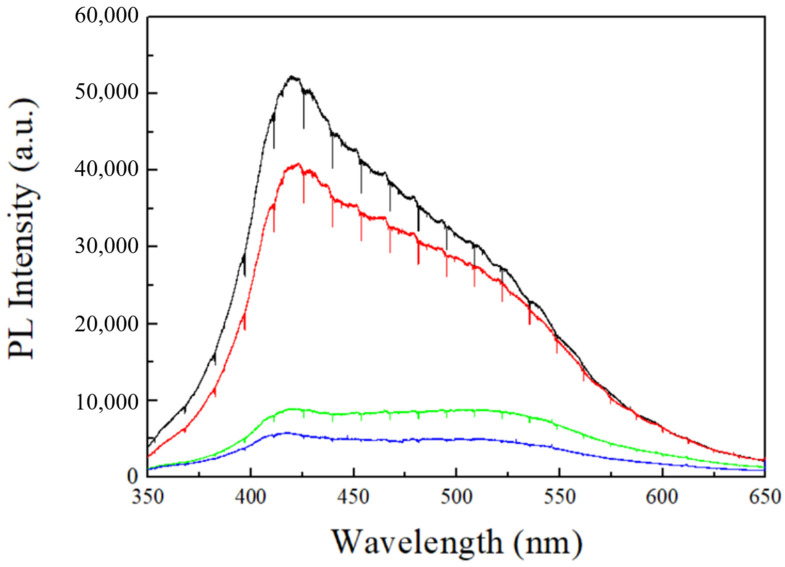
The PL spectrum from AAO fabricated at 40 V (black), 60 V (red), 80 V (green), and 100 V (blue) under the same thickness. It was collected and analyzed using a 325 nm laser with an integration time of 10 s.

The other important factor is the number of defects in AAO films under different conditions. In 2006, Lee et al. [21] proposed that the water content of MA is 0.3–0.4 wt.% in AAO, which is higher than the value of 0.1 wt.% in HA. Furthermore, it was also observed that the carbon content in MA is 2.4 wt.%, which is higher than the 1.8 wt.% in HA, and the reduction of these impurities significantly contributes to the increase in AAO hardness. In the anodization process with oxalic acid, carbon contents primarily originate from the oxalate ions in the solution. Therefore, observing the PL spectrum of AAO directly provides insights into the number of impurities. Figure 7 shows the PL spectra of AAO prepared at different voltages, with the PL intensities of 40 V (black), 60 V (red), 80 V (green), and 100 V (blue) arranged from high to low intensity. The PL spectra were collected and analyzed using a 325 nm laser with an integration time of 10 s. This demonstrates that AAO prepared at lower voltages exhibits stronger PL spectra. The PL characteristics of AAO were initially reported by Yamamoto et al. in 1981 [26], with a stronger signal in the PL spectrum near 470 nm, which is contributed by the oxalate ions in the AAO pore walls. In addition, Huang et al. [27] suggested that the PL in the AAO membrane originates from oxygen vacancies (F center) within the AAO membrane, and the emission peaks are located at 413 nm and 430 nm. Therefore, the PL properties of AAO prepared in oxalic acid involve these three peaks, resulting in a PL peak falling between 413–470 nm, consistent with our results for 40 V–60 V. However, at higher voltages, AAO exhibits an inconsistent PL spectrum. In 2007, Zheng et al. [31] reported a reduction in PL intensity as the anodization potential increases, with the peak shifting beyond 500 nm at 130 V. Their findings align closely with our PL measurements from high-voltage anodization of 80–100 V. The difference arises from their use of a low-temperature process and the addition of ethanol in the electrolyte to reduce Joule heating. In contrast, we utilized HPA for anodization at room temperature, allowing us to achieve even faster growth rates at 80 V and 100 V compared to their 130 V process. The decrease in PL intensity for high-voltage AAO can be well-explained by the concurrent improvement in surface hardness. Since PL of AAO originates from both oxalate ions and oxygen defects, these impurities in the AAO membrane can result in a decrease in its hardness. In the case of preparing AAO at high voltages, the rapid self-organization leads to a reduction in defects within the AAO pore wall, further enhancing the surface hardness with denser structure. The AAO hardness and PL peak intensities at 413 nm and 470 nm are listed in Table 2 for comparison. In Table 2, there is a negative correlation between AAO hardness and PL intensity. The PL intensities of 100 V AAO at 413 nm and 470 nm are 5629 and 4685, respectively, while the traditional mild anodized 40 V AAO exhibits significantly (8 times) higher intensities of 49,519 and 37,919. This indicates that defects such as anions and oxygen vacancies in AAO are much higher in MA conditions and indeed lead to a decrease in hardness. On the other hand, the hardness variation from 60 V to 80 V is most pronounced, increasing from 127 HV to 320 HV. This phenomenon is also reflected in the differences in PL intensity. The intensities of 60 V AAO at 413 nm and 470 nm are 37,365 and 32,389, respectively, while 80 V AAO has intensities of only 8315 and 8434, resulting in a difference by a factor of four. This also suggests that, when the voltage of AAO exceeds a certain degree, hardness suddenly increases, aligning with the abrupt increase in growth rate. The AAO prepared at 100 V exhibits the lowest PL intensity, with values of only 5629 at 413 nm and 4285 at 470 nm, indicating the highest hardness with the lowest impurities of anions and oxygen vacancies. Therefore, high-voltage anodization can effectively reduce impurities in AAO, while rapid self-organization and a dense structure are crucial to enhance surface hardness.

## 4. Conclusions

We successfully improved the surface hardness of AAO using a simple, cost-effective method: one-step anodization. Through an HPA approach to suppress Joule heating, the anodization voltage of AAO was increased to 80–100 V while preserving the complete pore structure of AAO without a burning effect. The growth rate is able to reach 1.9 μm/min at an anodization voltage of 100 V, which is a significant improvement compared to the 0.1 μm/min growth rate at 40 V. AAO produced under high-voltage parameters exhibited a denser structure due to rapid self-organization, resulting in fewer defects in water content, oxalate anions, and oxygen vacancies. Among samples with the same thickness (40 V–100 V) of about 12 μm, it was observed that the hardness of AAO at 100 V reached 423 HV, approximately 5 times higher than the traditional value of 83 HV at 40 V. The factors for the hardness enhancement of AAO at high voltage of 100 V are attributed to the decreased porosity and the reduction in defects such as water content, oxalate anions (carbon content), and oxygen vacancies in the AAO membrane. First, the reduction in porosity, as observed in SEM images, is linked to the increased voltage. The lower porosity indicates a decrease in the proportion of air within the AAO membrane, thereby increasing the overall structural strength. This contributes to the increased hardness of AAO produced under HA conditions. Second, the intensity observed in the PL spectrum decreases with increasing voltage, indicating a rapid reduction in anions and oxygen vacancies during high-voltage anodization. Moreover, the water content is also reduced by HA conditions. The decrease in defects in the AAO membrane leads to an increase of surface hardness.

## Figures and Tables

**Table 1:** The comparison of AAO parameters and pore morphology.

	Voltage (V)	Time (s)	Pore Diameter (nm)	Porosity (%)	Thickness (µm)
Sample 1	40	7200	31.1 ± 4.0	10.2 ± 0.9	11.9 ± 0.5
Sample 2	60	2520	33.1 ± 3.7	9.1 ± 0.8	12.2 ± 0.4
Sample 3	80	450	41.2 ± 3.7	7.3 ± 0.6	12.1 ± 0.6
Sample 4	100	390	43.3 ± 3.8	6.7 ± 0.5	12.3 ± 0.7

**Table 2:** The comparison of AAO surface hardness and PL intensity at 413 nm and 470 nm.

	Voltage (V)	Hardness (HV)	PL Intensity at 413 nm	PL Intensity at 470 nm
Sample 1	40	83	49,519	37,919
Sample 2	60	127	37,365	32,389
Sample 3	80	320	8315	8434
Sample 4	100	423	5629	4685

## Data Availability

Data that are presented in the coauthors’ research results and schematic drawing are available on request.
